# The Reliability and Validity of Current Technologies for Measuring Barbell Velocity in the Free-Weight Back Squat and Power Clean

**DOI:** 10.3390/sports8070094

**Published:** 2020-06-30

**Authors:** Steve W. Thompson, David Rogerson, Harry F. Dorrell, Alan Ruddock, Andrew Barnes

**Affiliations:** 1 Department of Sport and Physical Activity, Sheffield Hallam University, Sheffield S10 2BP, UK; d.rogerson@shu.ac.uk (D.R.); a.ruddock@shu.ac.uk (A.R.); a.barnes@shu.ac.uk (A.B.); 2 School of Sport and Exercise Science, University of Lincoln, Lincoln LN6 7TS, UK; hdorrell@lincoln.ac.uk

**Keywords:** velocity-based training, strength and conditioning, resistance training, load-velocity profile

## Abstract

This study investigated the inter-day and intra-device reliability, and criterion validity of six devices for measuring barbell velocity in the free-weight back squat and power clean. In total, 10 competitive weightlifters completed an initial one repetition maximum (1RM) assessment followed by three load-velocity profiles (40–100% 1RM) in both exercises on four separate occasions. Mean and peak velocity was measured simultaneously on each device and compared to 3D motion capture for all repetitions. Reliability was assessed via coefficient of variation (CV) and typical error (TE). Least products regression (LPR) (R^2^) and limits of agreement (LOA) assessed the validity of the devices. The Gymaware was the most reliable for both exercises (CV < 10%; TE < 0.11 m·s^−1^, except 100% 1RM (mean velocity) and 90‒100% 1RM (peak velocity)), with MyLift and PUSH following a similar trend. Poorer reliability was observed for Beast Sensor and Bar Sensei (CV = 5.1–119.9%; TE = 0.08–0.48 m·s^−1^). The Gymaware was the most valid device, with small systematic bias and no proportional or fixed bias evident across both exercises (R^2^ > 0.42–0.99 LOA = −0.03–0.03 m·s^−1^). Comparable validity data was observed for MyLift in the back squat. Both PUSH devices produced some fixed and proportional bias, with Beast Sensor and Bar Sensei being the least valid devices across both exercises (R^2^ > 0.00–0.96, LOA = −0.36–0.46 m·s^−1^). Linear position transducers and smartphone applications could be used to obtain velocity-based data, with inertial measurement units demonstrating poorer reliability and validity.

## 1. Introduction

Strong inverse linear relationships between load and velocity exist across many resistance-based exercises [1,2,3]. The facilitation and monitoring of training prescriptions, fatigue management, daily strength estimations, and motivation have all been proposed as benefits to implementing velocity-based measures in to practice [3,4,5]. However, the reliability and validity of some technologies such as linear position transducers (LPT), inertial measurement units (IMU), and smartphone applications designed to measure barbell velocity and facilitate the above still need further clarification in certain exercises. Moreover, comparisons between devices will provide coaches with practical recommendations on the most appropriate technology to utilize in the field.

Research has investigated the reliability of several devices in exercises such as the back squat, bench press, deadlift, and bench-pull [6,7,8,9]. LPTs (e.g., Gymaware, Tendo, Speed4Lifts, etc.) are typically considered the most appropriate tool for measuring barbell velocity in applied contexts [6,7,8,9,10,11]. LPTs utilize an optical encoder, tether-based system that enables the real-time collection of displacement-time data to calculate velocity [8]. A typical error (TE) of 6.0% to 8.9% has been observed in the Gymaware for mean and peak velocity across three trials at 80% 1RM in three common exercises [8]. Standard errors of measurement (SEM) of 3.9% to 9.9% in mean and peak velocity have also been observed in the back squat and bench press [11]. Other LPTs such as Speed4Lifts, T-Force, and Chronojump have also demonstrated good levels of reliability [7,9] leading to researchers utilizing these systems as criterion measures [10,12,13]. 

LPTs such as the Gymaware can be expensive (≈£2000) and are sometimes limited to barbell exercises. However, wearable or tether free IMUs can be more affordable and versatile within the training environment. IMUs rely on a combination of accelerometers and gyroscopes to measure acceleration data with respect to time [14], yet different devices can produce different results in terms of reliability. Large coefficient of variation (CV) (0.8% to 19.1%) and intraclass correlation coefficient (ICC) (0.46 to 0.99) ranges, as well as fixed and proportional bias, have been observed for the PUSH band across various exercises, equipment (smith-machine versus free-weight), metrics (mean and peak velocity), and methods (inter- versus intra-session and set reliability) [7,9,11,14,15,16]. IMUs such as the Beast Sensor and Bar Sensei are comparatively under-investigated [17,18], and require full validation against a criterion measure. Similarly, smartphone applications (e.g., MyLift) are new tools for measuring velocity and utilize the advanced technology in smartphones such as accelerometers, gyroscopes, and magnetometers [17,19]. Practically perfect ICCs (0.92 to 0.97) and low CVs (2.9% to 5%) in the back squat, bench press, and hip thrust exercises have been observed for the MyLift application (formerly PowerLift) [9,17,19], yet research in this area is still limited and further validation is therefore required.

Many studies have investigated the concurrent validity of LPTs, IMUs, or smartphone applications that utilize other LPTs as the criterion measurement [7,11,12,14,17,19]. This is is problematic given LPTs can still produce undesirable amounts of measurement error [7,9]. 3D motion capture is considered the gold-standard for measuring human movement due to its sophisticated technology, ability to measure all three planes of motion, small measurement error, and excellent repeatability [20,21]. Surprisingly, only two studies to date have employed 3D motion capture as the criterion measure when measuring barbell velocity [8,15]. Dorrell et al. [8] reported R^2^ values of 0.91 to 0.99 for peak and mean velocity across three exercises in the Gymaware, whereas Lake et al. [15] observed proportional bias in the PUSH in the bench press when employing least-products regression (LPR). Both studies, however, were limited to one or two loads, preventing analysis of the full load-velocity profile (LVP).

The majority of the literature in this area predominantly investigates strength-based exercises. Nevertheless, weightlifting exercises such as the power clean are also very common in strength and conditioning interventions given their favorable inter- and intra-day reliability and strong influence on physical skills such as jumping and sprinting [22,23]. The power clean, like other weightlifting derivatives, stimulates high force generation and impulse due to the requirement to lift heavy loads with high velocity [23,24]. Technical proficiency and consistency of such movements are integral to their impact on physical development [24,25] and thus perfecting this competency is essential. The reliability and validity of velocity-based devices when tracking such movements have, to date, never been investigated. 

Methodological errors and unrepresentative procedures such as the use of smith machine exercises, inaccurate estimations of relative loads, or limited evaluation across multiple sessions [7,9,10,14] have rendered much of the reliability and validity data in this area problematic, particularly within practical contexts. Moreover, exercises employed within this research tend to be limited to strength-based compound exercises. Assessments of reliability and validity of velocity-based devices used during explosive exercises and weightlifting derivatives such as the power clean are scarce, despite being prevalent in practice. Therefore, the aim of this research was to assess the inter-day and intra-device reliability and criterion validity of six common velocity-measuring systems (i.e., Gymaware, PUSH × 2, Bar Sensei, Beast Sensor, and MyLift) in competitive weightlifters in the free-weight back squat and power clean exercises. Whilst evidence for some of these technologies is scarce at the time of writing, we hypothesized that the Gymaware LPT would demonstrate the greatest reliability and validity of the devices, while the IMUs (i.e., PUSH, Bar Sensei, and Beast Sensor) and smartphone application (i.e., MyLift) would demonstrate lower levels of reliability and validity, comparatively.

## 2. Materials and Methods

### 2.1. Participants

In total, 10 healthy competitive weightlifters (mean ± SD; age: 25.0 ± 5.6 years, body mass: 73.6 ± 13.9 kg, stature: 169.6 ± 6.6 cm), with a minimum competition history of regional level within 12 months prior to data collection, regular weightlifting training, and strength levels of> 1.5 × body mass in the back squat, were recruited. A purposive sampling method was conducted given the specific population in question. The sample size was determined by the availability of this elite population and in conjunction with similar research in this area [14,17,19]. All participants were informed about the potential benefits and risks of the study before providing informed consent prior to data collection. Ethical approval was granted by the local institutions review board in accordance with the 7th revision (2013) of the declaration of Helsinki.

### 2.2. Study Design

The study assessed the reliability and validity of six velocity-based devices for measuring concentric mean and peak velocity against 3D motion capture in the free-weight back squat and power clean. Participants attended the laboratory on four separate occasions to complete an initial 1RM assessment, followed by three identical LVP sessions. All sessions were separated by 48–96 h to ensure adequate recovery time, with all data being collected systematically and independently. Velocity was recorded simultaneously on each device for each repetition. 

### 2.3. Methodology

During the baseline session, mass (kg) (InBody 720, Biospace, Seoul, Korea), stature (cm) (Harpenden, Holtain Ltd, Wales, UK), squat depth (cm), and power clean and back squat 1RMs were determined. The 1RM protocols were completed as per the National Strength and Conditioning Association (NSCA) guidelines [26]. Participants were habituated with the requirement to move loads with *‘maximal intent and velocity’*. The 1RM assessments consisted of incremental protocols (50–100% estimated 1RM) that culminated in the determination of the participant’s maximum load that could be lifted for one repetition. When participants reached the estimated 1RM, loads were increased by 1 kg–5 kg in order to find a true 1RM for each individual. A maximum of five attempts were allowed at 1RM and rest periods between sets were three to five minutes. A calibrated, International Weightlifting Federation (IWF) approved 20 kg bar and bumper plates (Werksan, Akyurt, Turkey) were used throughout this study. Participants undertook a standardized, individualized warm up protocol consisting of 5 min of cycling at 100 W (Ergomedic 874E, Monark, Vansbro, Sweden) and a combination of mobility, dynamic flexibility, and light barbell work.

The three subsequent visits were procedurally identical. Following completion of the standardized warm up, participants completed incremental load assessments ranging from 40–100% 1RM (10% increments) in the power clean and back squat. Throughout the protocol, participants performed 3 repetitions for light loads (≤60%), 2 repetitions for moderate loads (70–80%), and 1 repetition for heavy loads (≥90%), with three to five-minute rest periods between sets [6]. All concentric portions of each repetition were instructed to be performed with *‘maximal intent and velocity’* to maximize the reliability of the movement. International Powerlifting Federation and IWF technical and competition rules and regulations guidelines were adhered to for the back squat and power clean movements, respectively [27,28]. The bar was placed in a high-bar position during the back squat and was situated on the superior aspect of the trapezius muscles. A lift was deemed successful when the greater trochanter was situated inferior to the lateral epicondyle of the knee at the lowest point of descent and the individual was able to fully extend the lower limbs on ascent. A power clean was deemed successful if the bar was caught across the glenohumeral joints and the participant could fully extend the lower extremities to finish the lift, and the greater trochanter finished superior to the lateral epicondyle of the knee at the lowest point of displacement during the catch phase. All lifts were assessed by an accredited strength and conditioning (S-C) coach and retrospectively via a smartphone camera system (iPhone 7, iOS 11.4.1, Apple, Cupertino, CA, USA).

### 2.4. Equipment Setup

A 12 camera, 3D motion capture (Raptor, Motion Analysis Cooperation, Rohnert Park, CA, USA) setup was used as the criterion measure, sampling at 250 Hz and recording three-dimensional time-displacement data. The capture volume was central to the setup, with the cameras evenly spaced around it. A full calibration was performed prior to each session, with a measurement error of <0.3 mm accepted [8]. Retro-reflective markers were placed on either end of the barbell in order to create a virtual mid-point on which velocity measurements were based [8].

The six devices tested can be seen in Table 1. A fourth generation iPad mini (Apple, Cupertino, CA, USA) ran the software for each IMU and LPT device (iOS 11.4.1), with an iPhone 7 used for the smartphone app. The MyLift smartphone application and beast sensor IMU were only used for the back squat due to the capabilities of the two devices at the time of data collection. The MyLift application required an input of descent displacement prior to each repetition, which was measured for each individual during the baseline visit.

### 2.5. Data Processing

Positional marker data were identified, rectified, and gapped-filled using the Cortex software (v5.3.3, Motion Analysis Corporation, Rohnert Park, CA, USA) before being analyzed using custom-written MATLAB codes (R2017a, MathWorks, Natick, MA, USA). A residual analysis of marker displacement (2 Hz to 13 Hz) was performed from a sample of data to determine the most appropriate cut-off filtering frequency for both lifts. Following this, a zero lag, second-order Butterworth low-pass filter was applied to the data, with a cut-off frequency of 6 Hz for back squat and 8 Hz for power clean. A barbell virtual midpoint was created by taking the mean of the diametric markers. 

Vertical velocity of the concentric phase was calculated for the virtual midpoint position. The finite central difference method was employed for the differentiation of marker data [29]. The lowest and highest point of vertical displacement defined the start and end of the concentric phase for the back squat, respectively [8,15]. The start of the power clean was defined as the first instance of positive vertical velocity coinciding with the first instance of vertical displacement. The end of the power clean was defined as the highest point of vertical displacement that coincided with the last instance of positive velocity, representing the initial point of contact during the catch phase [24,25]. Peak and mean velocity for each repetition were subsequently determined from the instantaneous highest velocity value and by averaging the velocity data over the pre-defined concentric phase, respectively [8,15]. All devices used in this study were operated as per the manufacturer’s instructions and all directly measured or calculated barbell velocity (Table 1).

### 2.6. Statistical Analysis

Univariate and multivariate outliers and skewness and kurtosis analyses were performed to determine if the data were normally distributed. Data were analyzed using SPSS 24.0 (IBM, New York, NY, USA) and a custom-built spreadsheet (Microsoft Excel, Microsoft, Albuquerque, NM, USA) [30]. The validity of the experimental devices was assessed against the criterion measure using LPR, expressed as an R^2^ value, in conjunction with 95% confidence intervals (CI) of the slope and intercept to assess fixed and proportional bias [8,15,31,32]. Moreover, 95% limits of agreement (LOA) were used to assess for systematic bias between the criterion and the other devices. Inter-day and intra-device reliability were analyzed using a combination of TE and CV.

## 3. Result

Data was collected for 10 participants across three repeat sessions. Each back squat LVP consisted of 10 incremental loads, with nine being completed for the power clean. This totaled 57 pieces of data per participant and 570 data points for the total sample.

### 3.1. Reliability

#### 3.1.1. Back Squat

Gymaware and MyLift produced the highest levels of reliability in the back squat (Table 2). However, CVs and TEs typically increased as relative load increased. CVs > 10% were observed for 90% 1RM (peak velocity) and 100% 1RM (mean and peak velocity) for the Gymaware, and 90% and 100% 1RM (mean velocity) for MyLift (Table 2). Comparable CV data was evident for PUSHbody (40% 1RM to 80% 1RM) and PUSHbar (40% 1RM to 70% 1RM). However, larger TEs were typically observed. Larger CVs and TEs were evident for the heavier loads (90% to 100% 1RM) for both PUSH devices compared with Gymaware and MyLift for mean velocity, with comparable or slightly lower CVs evident in peak velocity. Larger CVs and TEs were observed for both Bar Sensei and Beast Sensor across most relative loads compared to the other technologies (Table 2). Typically, TEs and CVs were smaller for mean velocity compared to peak velocity for all devices.

#### 3.1.2. Power Clean

The Gymaware produced smaller CVs and TEs across all relative loads compared to the PUSH and Bar Sensei devices (Table 2). Light and moderate loads (40% to 70% 1RM) for PUSHbody displayed comparable CVs and TEs to the Gymaware, yet heavier loads (>80% 1RM) were higher in both velocity metrics. Larger CVs and TEs were observed for the PUSHbar for both mean and peak velocity. Bar Sensei had the least favorable reliability data for mean velocity but was comparable to the Gymaware for peak velocity.

### 3.2. Validity

#### 3.2.1. Back Squat

**Figure 1:**
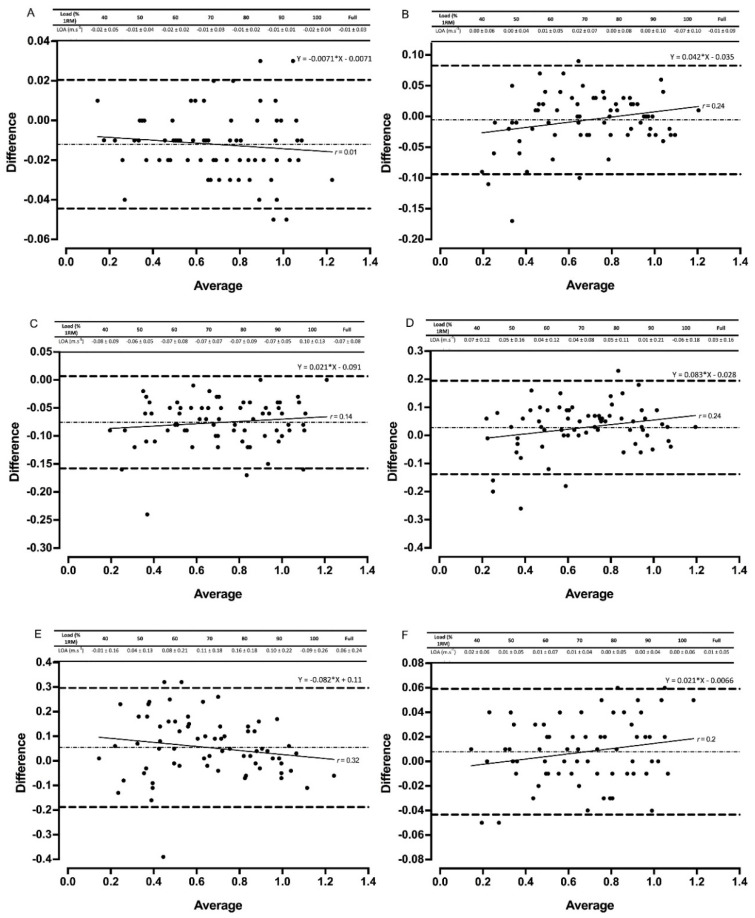
Bland–Altman plots exhibiting variation in velocity-based devices versus 3D motion capture for full datasets for mean velocity in the back squat. The mean systematic bias (dash-dot line) and 95% confidence intervals (dashed line) are displayed with the regression line (solid line) and the *r* value. The table above displays the limits of agreement (LOA) ± 95% confidence intervals for each relative load. Gymaware (**A**), PUSHbody (**B**), PUSHbar (**C**), Bar Sensei (**D**), Beast Sensor (**E**), and MyLift (**F**) devices are all shown.

**Figure 2:**
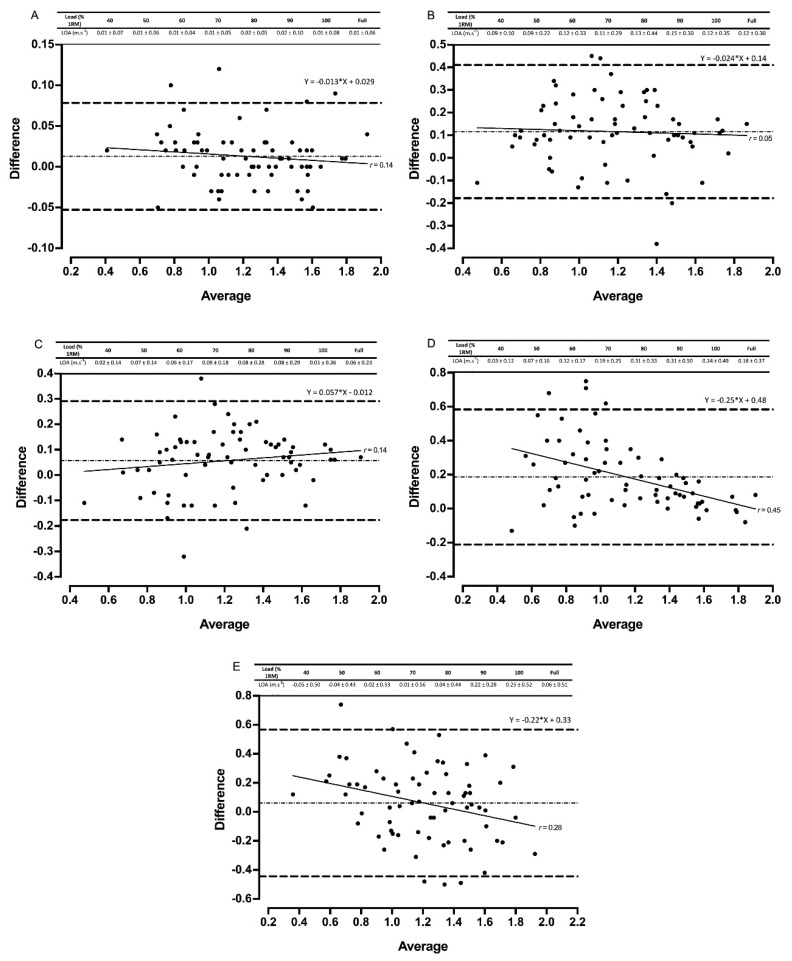
Bland–Altman plots exhibiting variation in velocity-based devices versus 3D motion capture for full datasets for peak velocity in the back squat. The mean systematic bias (dash-dot line) and 95% confidence intervals (dashed line) are displayed with the regression line (solid line) and the *r* value. The table above displays the limits of agreement (LOA) ± 95 % confidence intervals for each relative load. Gymaware (**A**), PUSHbody (**B**), PUSHbar (**C**), Bar Sensei (**D**), and Beast Sensor (**E**) devices are all shown.

Gymaware demonstrated the strongest validity when compared to 3D motion capture during the back squat (Table 3, Figure 1 and Figure 2). No fixed or proportional bias was observed for either device when measuring mean or peak velocity for the back squat, with R^2^ values ≥ 0.95. The MyLift application followed a similar trend, with R^2^ values ≥ 0.88 (Table 3, Figure 1). Small systematic bias was also evident for the two devices for both mean and peak velocity (Figure 1 and Figure 2). 

The two PUSH devices produced R^2^ values of 0.80 to 0.97 as revealed from the LPR (Table 3). Fixed and proportional bias was evident for loads of 60%, 80%, 90% 1RM and 80% 1RM for mean and peak velocity, respectively. Peak velocity was over-estimated in both devices as shown by the LOAs (Figure 1 and Figure 2). Smaller mean systematic bias was evident in the PUSHbody than the PUSHbar for mean velocity (Figure 1 and Figure 2).

Fixed and proportional bias was evident across the full datasets for the Beast Sensor (mean and peak velocity) and Bar Sensei (peak velocity only) (Table 2). Systematic bias was also present for both devices across both velocity metrics (Figure 1 and Figure 2).

#### 3.2.2. Power Clean

**Figure 3:**
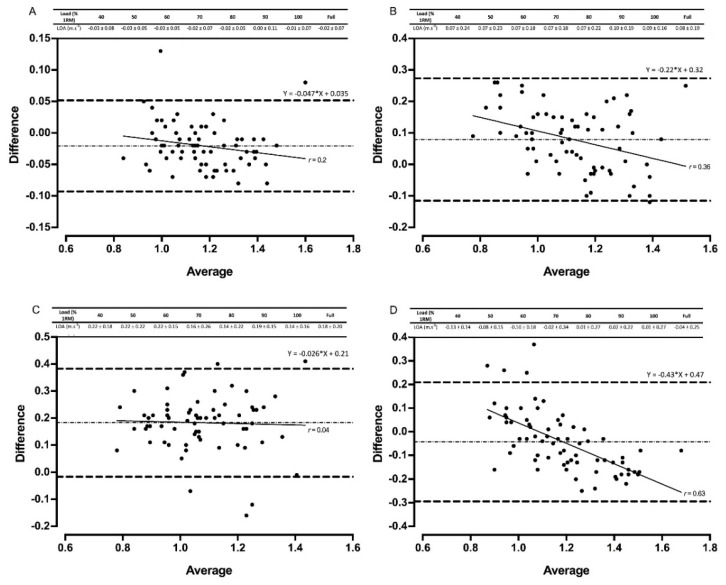
Bland–Altman plots exhibiting variation in velocity-based devices versus 3D motion capture for full datasets for mean velocity in the power clean. The mean systematic bias (dash-dot line) and 95% confidence intervals (dashed line) are displayed with the regression line (solid line) and the *r* value. The table above displays the limits of agreement (LOA) ± 95 % confidence intervals for each relative load. Gymaware (**A**), PUSHbody (**B**), PUSHbar (**C**), and Bar Sensei (**D**) devices are all shown.

**Figure 4:**
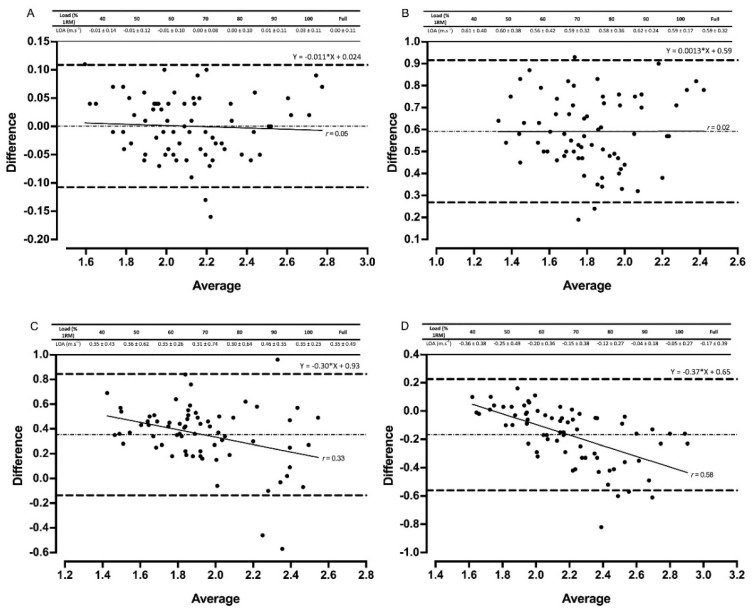
Bland–Altman plots exhibiting variation in velocity-based devices versus 3D motion capture for full datasets for peak velocity in the power clean. The mean systematic bias (dash-dot line) and 95% confidence intervals (dashed line) are displayed with the regression line (solid line) and the *r* value. The table above displays the limits of agreement (LOA) ± 95 % confidence intervals for each relative load. Gymaware (**A**), PUSHbody (**B**), PUSHbar (**C**), and Bar Sensei (**D**) devices are all shown.

Of the four devices that were used to assess the validity of the power clean exercise, the Gymaware demonstrated the strongest validity when compared to the criterion measure. No fixed or proportional bias was observed, with R^2^ values > 0.75 for all relative loads apart from 90% and 100% 1RM for mean velocity (Table 4). Mean velocity was slightly underestimated in the Gymaware, but no systematic bias was present when measuring peak velocity (Figure 3 and Figure 4). The three IMUs displayed a fixed and proportional bias across multiple relative loads and the full datasets for both mean and peak velocity (Table 4). PUSHbar was the only IMU to demonstrate no fixed bias across any of the relative loads and full dataset. Both PUSH devices overestimated mean (up to 0.26 m·s^−1^) and peak (up to 0.74 m·s^−1^) velocity, whilst Bar Sensei underestimated both velocity measurements (up to 0.36 m·s^−1^) (Figure 3 and Figure 4). 

## 4. Discussion

The aim of this research was to assess the day-to-day reliability and criterion validity of six systems that measure barbell velocity in the free-weight back squat and power clean. The major findings of this research are as follows: (1) Gymaware was the most reliable and valid tool for both exercises when measuring mean and peak velocity; (2) MyLift produced comparable validity and reliability results to the Gymaware; (3) PUSH devices demonstrated good levels of validity and reliability, despite detectable measurement error; (4) Bar Sensei and Beast Sensor exhibited poor reliability and validity.

Gymaware demonstrated the greatest reliability and validity when measuring mean and peak velocity. Practically perfect R^2^ values, small systematic bias, and no fixed or proportional bias were evident across all loads, in addition to favorable levels of between-session reliability for all back squat loads except 100% 1RM (Figure 1, Figure 2, Figure 3 and Figure 4, Table 2, Table 3 and Table 4). Our data supports previous research that compares Gymaware to 3D motion capture wherein R^2^ values of 0.99, mean differences (± SD) of 0.01 m·s^−1^ ± 0.01 m·s^−1^ and −0.02 m·s^−1^ ± 0.03 m·s^−1^, and between-trial CVs of 7.0% to 8.1% were observed in the free-weight back squat [8]. However, data was limited to 80% 1RM only. In comparison, our study provides practitioners with useable reliability and validity data across a full LVP in 10% increments. Research elsewhere indicates that Gymaware is valid when contrasted with sophisticated LPT systems [6] and reliable across testing occasions [11]. Despite this, we observed poorer R^2^ values during heavier loads in the power clean for mean velocity (Table 3), suggesting that the complexities and 3D nature of weightlifting exercises might limit the application of LPTs. LPTs use a rotary or optical encoder attached to a tether designed to produce time-displacement data in order to calculate velocity and account for varying angles of tether retraction by applying trigonometry, limiting potential error in the measurement and processing of velocity [6,8,33]. Therefore, our data, in conjunction with previous literature, indicates that the Gymaware is a reliable and valid tool for measuring barbell velocity.

MyLift demonstrated comparable validity data to Gymaware for mean velocity in the back squat (Figure 1, Table 2 and Table 3). Our study is the only one to compare MyLift against 3D motion capture and assess fixed, proportional, and systematic bias, making comparisons with previous research difficult. However, Perez-Castilla et al. [9] assessed the application against an infrared motion sensing system and found only small systematic bias, but did observe heteroscedasticity. Additionally, research adopting more common validity statistics observed practically perfect correlation coefficients (*r* = 0.94; SEE = 0.03; ICC = 0.97) when compared with an LPT [17,19]. CVs and TEs indicated good between-session reliability for loads of 40% to 80% 1RM in our study, supporting recent research (ICC = 0.96 to 0.98; CV = 2.85% to 4.97%) [9,17]. Conversely, poor levels of reliability (CV > 20%) have been observed in the bench press [7], perhaps due to observer error and improper usage [34]. Therefore, if operated by an experienced professional that understands the specific nuances of different exercises, this device could be an effective tool for practitioners. Future research should further investigate the inter-rater reliability using similar devices to determine if prior knowledge of the exercises is an important pre-requisite.

Good levels of reliability were observed for the back squat except for 90% and 100% 1RM loads in three of the six devices (Gymaware, MyLift, and PUSHbody). This was supported by between-session reliability data from the criterion measure (CVs = 9.0% to 21.5%; TEs = 0.03 m·s^−1^ to 0.14 m·s^−1^). However, further analysis of the LOA at 100% 1RM indicated high, consistent levels of agreement between the aforementioned devices and criterion measure. For example, the mean velocity of the Gymaware at this load indicated a difference between the mean systematic bias data of 0.01 m·s^−1^ (−0.01 m·s^−1^ to 0.00 m·s^−1^) over the three sessions. Moreover, it has recently been suggested that heavy loads can alter limb and torso displacement, joint moments, and joint angular velocities, resulting in variability in barbell kinematics [35,36]. Therefore, it could be suggested that the poor reliability data at these heavier loads could be a result of human movement variability, as opposed to the devices. Reliability data for the power clean also supports this suggestion, particularly in the Gymaware. CVs of <5% at all but one load indicates the between-session reliability of this device is very strong. This could be because of a greater technical competency required to achieve key positions in this lift [24]. By perfecting the lifts using a technical model [24], the consistency of torso and limb positioning could have a positive influence on the consistency of barbell kinematics, such as velocity. 

Both PUSH band devices experienced good validity and reliability in the back squat (Figure 3 and Figure 4, Table 3 and Table 4). However, fixed and proportional bias was evident in some loads (Table 3) as well as positive and/or negative mean systematic bias (−0.08 m·s^−1^ to 0.15 m·s^−1^), with 95% CI as high as 0.44 for peak velocity. This suggests that PUSH may under- or overestimate velocity data, which could be problematic in practice. Assessing PUSH’s validity using LPR has only occurred once in the literature, in which proportional bias was also evident at 60% and 90% 1RM for mean velocity in the free-weight bench press [15]. This suggests that the PUSH IMU may not be fully valid and practitioners should be aware of the LOA if utilizing in their practices. Both PUSH devices, however, did demonstrate favorable reliability for the light and moderate loads in the back squat, as well as the PUSHbody in the power clean, supporting research that has reported the presence of heteroscedasticity across sessions [13]. Practitioners, therefore, might need to reconsider previously reported recommendations when employing PUSH and take into consideration the device’s ability to accurately estimate mean and peak velocity [3,14,15].

The poorest validity and reliability data were observed for Beast Sensor and Bar Sensei (Figure 1, Figure 2, Figure 3 and Figure 4, Table 2, Table 3 and Table 4). Large CVs and TEs, mean systematic biases and fixed and proportional biases were evident for both technologies across the majority of relative loads, supporting that of previous research [9,18]. Similarly, fixed, proportional and systematic bias was evident for all three IMUs assessed during the power clean, suggesting that IMUs may not be appropriate for measuring technical and explosive exercises. The ‘black-box’ technology sometimes employed by IMU manufacturers could contribute to the poor levels of reliability and validity, limiting the option to factor in measurement error [15]. Some IMUs (e.g., PUSHbody, Beast Sensor) have been designed as wearable, being placed on the forearm, for example, which might offer an explanation for the measurement error observed, particularly in the power clean where movements around the elbow could create variability in the velocity metrics being reported [9,13]. Data, therefore, suggests more developments are needed for IMUs to be fully valid and reliable [6,9,13,18].

Affordability and practicality are important in practice. Despite Gymaware performing better across exercises, the device is considerably more expensive than MyLift, for example (≈ £1940 more). Additionally, MyLift allows for manual detection of the concentric phase, potentially reducing the chance of erroneous errors common to ‘black-box’ technology. However, this method limits exercise choice and prevents the use of peak velocity. Similarly, the analysis of videos after the termination of a set also prevents immediate bio-feedback that has shown to be advantageous during resistance training [5]. The wearable nature of some IMUs allows for greater flexibility of usage. Practitioners need to consider associated costs, ease of use and accuracies prior to determining the most appropriate technology to purchase.

While this study has provided some useful practical insights regarding the use of VBT technologies, its limitations must be presented. Given that each system utilizes different technical configurations (e.g., sampling rate, rep detection, technology, etc.), a true comparison for each device to the criterion measure was difficult. Nevertheless, our definition of the concentric phase for each lift when using the criterion measure was in accordance with previous research [8,15]. Equally, given the exercise-specific nature of the LVP [6,37], the generalizability of our data may be limited. Finally, inter- and intra-device translation is poor, preventing generalizing our data to other LPTs, IMUs, or smartphone applications.

## 5. Conclusions

This is the first study to compare multiple systems against a true criterion measure and was the first to investigate a weightlifting derivative. Our results demonstrate that the Gymaware was the most valid and reliable device for the back squat and power clean exercises, closely followed by MyLift for the back squat. IMUs should be used with caution, especially with heavier loads given the over- or under-estimation of mean and peak velocity. However, PUSH demonstrated good reliability for loads up to 80% 1RM. LPTs offered the greatest levels of reliability and validity but are expensive and limited to barbell exercises. IMUs and iPhone applications are more affordable but can be limited in validity.

## 6. Practical Applications

Velocity-based methods for prescription (i.e., load-manipulation [38,39] and volume control [40]), testing, and monitoring (i.e., load-velocity profiling [37]) are frequently applied to sport science support and research. Practitioners, therefore, must be confident in the technology they use to ensure effective implementation. The reliability and validity of such technologies is of high importance for coaches and thus the findings of this research provide useful advice into the most appropriate devices to employ. Based on our data, LPTs such as the Gymaware should be implemented where possible given their superior reliability and validity across exercises; practicalities, and budget permitting. If available funding is limited, iPhone applications such as MyLift are suitable alternatives, yet they are restricted in their application across exercises. Similarly, some IMUs (PUSH) can offer reliable data, but practitioners must be aware of the measurement error associated with them. The implementation of velocity-based technology into S-C practice is effective if employing reliable devices. However, ease of use, software usability, economic cost, and durability also need to be taken in to account. 

## Figures and Tables

**Table 1:** The specifications of each velocity-based device.

Device	Type	Technology	Cost (£)	Sampling Rate	Location of Device
Gymaware	Linear position transducer	Optical encoder (records displacement-time curve data in order to determine changes in bar position)	£1950	20 millisecond time points down-sampled to 50 Hz	Tether attachment 100 mm from the end of the right hand-side of the barbell
Bar Sensei	Inertial measurement unit	Not reported	£305 approx.	Not reported	Placed on the left-hand side of the barbell, directly inside the collar using the sleeve provided by the manufacturer
PUSHbody (PUSH band located on the forearm)	Wearable inertial measurement unit	3-axis accelerometer and gyroscope providing 6 degrees of freedom in coordinate system. (Integration of acceleration data with respect to time)	£250 approx.	1000 Hz down-sampled to 200 Hz	Worn on the right forearm immediately inferior to the elbow crease with the on/off button located proximally as suggested by the manufacturer
PUSHbar (PUSH band located on the barbell)	Inertial measurement unit	3-axis accelerometer and gyroscope providing 6 degrees of freedom in coordinate system. (Integration of acceleration data with respect to time)	£250 approx.	1000 Hz down-sampled to 200 Hz	Placed on the right hand-side of the barbell, directly inside the collar using the bar sleeve provided by the manufacturer
Beast Sensor	Wearable inertial measurement unit	3-axis accelerometer, gyroscope and magnetometer. (Integration of vertical acceleration data with respect to time)	£250 approx.	50 Hz	Worn on the superior aspect of the right wrist using a wrist band provided by the manufacturer
MyLift (PowerLift at the time of data collection)	Smartphone application	Manual frame-by-frame inspection of slow-motion video. (Pre-defined range of motion/time inputted prior to data collection)	£9.99	240 Hz (720p video quality)	Located directly behind the participant and fixed in position using a tripod in order for the full barbell to be visible on the iPhone 7 (iOS 11.4.1) screen

**Table 2:** Test-retest reliability data for six devices in the back squat and power clean.

Load (%)	Back Squat				Power Clean			
	TE (m·s^−1^)		CV (%)		TE (m·s^−1^)		CV (%)	
	MV	PV	MV	PV	MV	PV	MV	PV
Gymaware								
40	0.04	0.08	4.5	5.6	0.05	0.09	3.6	3.7
50	0.03	0.07	3.4	4.9	0.03	0.08	2.2	3.7
60	0.02	0.08	2.9	6.0	0.03	0.07	2.4	3.1
70	0.03	0.10	4.5	8.3	0.04	0.05	3.2	2.5
80	0.04	0.09	7.0	8.6	0.04	0.08	3.3	3.8
90	0.04	0.09	9.5	12.6	0.08	0.07	8.9	3.9
100	0.03	0.15	13.6	22.0	0.04	0.06	4.3	4.0
Full	0.04	0.10	9.8	11.3	0.05	0.07	4.9	3.3
PUSH Body								
40	0.03	0.09	3.5	6.0	0.06	0.08	4.9	4.9
50	0.04	0.15	4.1	9.9	0.06	0.08	5.2	5.2
60	0.04	0.11	5.4	9.1	0.05	0.08	4.5	4.5
70	0.03	0.10	5.0	8.9	0.09	0.12	7.7	7.7
80	0.03	0.07	5.2	6.8	0.10	0.14	10.2	10.2
90	0.07	0.11	15.6	11.0	0.09	0.13	11.3	11.3
100	0.04	0.08	14.9	11.4	0.09	0.12	11.4	11.4
Full	0.05	0.11	10.6	11.3	0.08	0.11	8.3	8.3
PUSH Bar								
40	0.06	0.09	5.2	5.7	0.20	0.36	21.5	21.5
50	0.08	0.10	9.2	7.5	0.18	0.33	19.0	17.9
60	0.05	0.12	5.1	9.4	0.17	0.42	18.9	25.4
70	0.04	0.09	5.9	8.3	0.13	0.22	14.6	13.4
80	0.09	0.09	14.3	8.8	0.14	0.25	16.3	15.6
90	0.09	0.12	20.3	14.2	0.15	0.31	18.1	22.2
100	0.06	0.09	15.4	11.6	0.10	0.23	13.3	17.5
Full	0.07	0.11	14.5	11.0	0.21	0.32	18.6	20.5
Bar Sensei								
40	0.08	0.14	9.1	9.4	0.23	0.20	20.4	7.7
50	0.09	0.10	13.5	7.6	0.16	0.15	13.8	6.5
60	0.07	0.08	8.8	8.0	0.13	0.13	12.1	5.8
70	0.07	0.09	10.7	10.2	0.13	0.19	11.8	8.8
80	0.08	0.24	18.3	35.8	0.13	0.13	14.9	6.1
90	0.08	0.12	19.1	18.0	0.15	0.14	17.7	7.9
100	0.13	0.12	60.5	28.5	0.14	0.15	18.4	8.5
Full	0.09	0.13	22.1	18.7	0.16	0.17	15.9	8.7
Beast Sensor								
40	0.05	0.10	5.1	6.2	-	-	-	-
50	0.06	0.11	7.6	7.4	-	-	-	-
60	0.08	0.15	12.0	11.8	-	-	-	-
70	0.12	0.27	22.4	25.8	-	-	-	-
80	0.22	0.33	72.3	54.0	-	-	-	-
90	0.21	0.48	75.8	119.9	-	-	-	-
100	0.15	0.29	40.6	65.1	-	-	-	-
Full	0.14	0.30	42.4	53.2	-	-	-	-
MyLift								
40	0.04	-	4.2	-	-	-	-	-
50	0.03	-	3.7	-	-	-	-	-
60	0.04	-	5.5	-	-	-	-	-
70	0.03	-	4.9	-	-	-	-	-
80	0.04	-	6.8	-	-	-	-	-
90	0.05	-	12.6	-	-	-	-	-
100	0.03	-	13.8	-	-	-	-	-
Full	0.05	-	9.7	-	-	-	-	-

*%*, percentage of one repetition maximum; CV, coefficient of variation; MV, mean velocity; PV, peak velocity; TE, typical error.

**Table 3:** Least products regression for 6 devices in the back squat in comparison to 3D motion capture.

Load (%)	R^2^		Slope (95% CI)		Intercept (95% CI)	
	MV	PV	MV	PV	MV	PV
Gymaware						
40	0.95	0.97	1.010 (0.824, 1.196)	1.033 (0.896, 1.170)	−0.027 (−0.221, 0.167)	−0.042 (−0.263, 0.178)
50	0.95	0.98	0.990 (0.808, 1.172)	0.971 (0.866, 1.075)	−0.004 (−0.173, 0.166)	0.054 (−0.100, 0.209)
60	0.98	0.99	1.046 (0.931, 1.160)	0.983 (0.908, 1.059)	−0.056 (−0.152, 0.040)	0.031 (−0.073, 0.136)
70	0.97	0.99	1.073 (0.917, 1.229)	0.957 (0.870, 1.044)	−0.060 (−0.172, 0.052)	0.067 (−0.040, 0.174)
80	0.99	0.99	0.990 (0.892, 1.088)	0.979 (0.885, 1.073)	−0.002 (−0.061, 0.058)	0.040 (−0.064, 0.144)
90	0.99	0.96	1.014 (0.943, 1.084)	0.910 (0.761, 1.059)	−0.014 (−0.048, 0.020)	0.110 (−0.040, 0.261)
100	0.97	0.97	0.965 (0.826, 1.104)	0.936 (0.813, 1.059)	−0.001 (−0.042, 0.039)	0.066 (−0.043, 0.174)
Full	0.99	0.99	0.991 (0.979, 1.004)	0.970 (0.931, 1.009)	−0.005 (−0.014, 0.003)	0.054 (−0.003, 0.110)
PUSH Body						
40	0.92	0.94	0.968 (0.730, 1.207)	1.026 (0.819, 1.233)	0.031 (−0.214, 0.275)	0.051 (−0.265, 0.368)
50	0.96	0.76	0.975 (0.816, 1.134)	0.852 (0.459, 1.245)	0.022 (−0.125. 0.168)	0.291 (−0.261, 0.844)
60	0.95	0.45	0.813 (0.659, 0.967) †	0.648 (0.069, 1.227)	0.160 (0.035, 0.285) *	0.562 (−0.170, 1.294)
70	0.88	0.60	0.842 (0.594, 1.090)	0.723 (0.238, 1.208)	0.128 (−0.043, 0.300)	0.425 (−0.127, 0.976)
80	0.92	0.37	0.746 (0.569, 0.922) †	0.482 (−0.033, 0.997) †	0.153 (0.048, 0.259) *	0.638 (0.114, 1.162) *
90	0.79	0.53	0.624 (0.365, 0.883) †	0.912 (0.217, 1.607)	0.173 (0.048, 0.298) *	0.227 (−0.380, 0.835)
100	0.58	0.48	0.790 (0.247, 1.333)	1.320 (0.195, 2.445)	0.004 (−0.184, 0.193)	−0.144 (−0.956, 0.727)
Full	0.97	0.80	1.028 (0.984, 1.072)	0.994 (0.918, 1.070)	−0.025 (−0.057, 0.007)	0.133 (0.053, 0.213)
PUSH Bar						
40	0.69	0.91	1.224 (0.835, 1.613)	1.185 (0.894, 1.476)	−0.326 (−0.755, 0.102)	−0.271 (−0.735, 0.193)
50	0.95	0.89	1.000 (0.814, 1.186)	1.063 (0.763, 1.362)	−0.062 (−0.245, 0.120)	−0.019 (−0.444, 0.405)
60	0.83	0.84	0.757 (0.479, 1.036)	0.881 (0.574, 1.188)	0.143 (−0.106, 0.393)	0.212 (−0.196, 0.621)
70	0.84	0.80	1.082 (0.691, 1.473)	1.091 (0.655, 1.528)	−0.135 (−0.440, 0.170)	−0.019 (−0.525, 0.487)
80	0.87	0.60	0.745 (0.505, 0.985) †	0.818 (0.271, 1.365)	0.099 (−0.061, 0.259)	0.267 (−0.308, 0.842)
90	0.92	0.56	0.858 (0.657, 1.059)	0.951 (0.257, 1.644)	0.010 (−0.098, 0.118)	0.128 (−0.525, 0.781)
100	0.39	0.41	0.661 (−0.013, 1.335)	0.890 (0.027, 1.752)	0.023 (−0.236, 0.282)	0.100 (−0.652, 0.851)
Full	0.97	0.86	1.010 (0.969, 1.050)	0.982 (0.888, 1.076)	−0.082 (−0.115, −0.050) *	0.078 (−0.037, 0.193)
Bar Sensei						
40	0.82	0.96	0.702 (0.437, 0.968) †	0.840 (0.051, 0.509) †	0.352 (0.098, 0.607) *	0.280 (0.051, 0.509) *
50	0.75	0.93	0.599 (0.313, 0.885) †	0.784 (0.605, 0.962) †	0.394 (0.143, 0.645) *	0.377 (0.123, 0.631) *
60	0.67	0.82	0.660 (0.285, 1.035)	0.968 (0.599, 1.336)	0.303 (0.009, 0.597) *	0.163 (−0.302, 0.628)
70	0.86	0.66	0.778 (0.520, 1.035)	0.825 (0.346, 1.303)	0.188 (0.015, 0.361) *	0.372 (−0.135, 0.880)
80	0.66	0.52	0.871 (0.362, 1.380)	0.680 (0.144, 1.217)	0.121 (−0.156, 0.398)	0.566 (0.120, 1.012) *
90	0.23	0.10	0.309 (−0.147, 0.765) †	0.332 (−0.470, 1.133)	0.326 (0.112, 0.540) *	0.777 (0.191, 1.362) *
100	0.01	0.02	0.359 (−0.233, 0.950) †	0.132 (−0.807, 0.171) †	0.147 (−0.056, 0.349)	0.737 (0.171, 1.303) *
Full	0.87	0.80	1.028 (0.945, 1.111)	0.712 (0.625, 0.799) †	0.010 (−0.048, 0.068)	0.485 (0.386, 0.583) *
Beast Sensor						
40	0.64	0.10	0.646 (0.250, 1.042)	0.319 (−0.442, 1.080)	0.354 (−0.058, 0.765)	1.079 (−0.193, 2.351)
50	0.71	0.12	0.719 (0.347, 1.091)	0.470 (−0.590, 1.531)	0.282 (−0.048, 0.612)	0.763 (−0.849, 2.375)
60	0.49	0.00	0.414 (0.067, 0.761) †	0.038 (−0.857, 0.933) †	0.511 (0.250, 0.771) *	1.320 (0.100, 2.539) *
70	0.46	0.00	0.499 (0.062, 0.936) †	−0.097 (−1.043, 0.848) †	0.406 (0.139, 0.673) *	1.353 (0.165, 2.541) *
80	0.58	0.02	0.499 (0.154, 0.844) †	0.345 (−1.716, 2.407)	0.377 (0.224, 0.531) *	0.742 (−1.716, 2.952)
90	0.12	0.58	0.271 (−0.172, 0.713) †	0.927 (0.281, 1.574)	0.368 (0.199, 0.538) *	0.273 (−0.252, 0.798)
100	0.20	0.15	0.236 (−0.146, 0.619) †	0.385 (−0.357, 1.127)	0.188 (0.041, 0.335) *	0.626 (0.141, 1.110) *
Full	0.80	0.57	0.835 (0.736, 0.933) †	0.622 (0.493, 0.751) †	0.159 (0.092, 0.227) *	0.506 (0.346, 0.667) *
MyLift						
40	0.96	-	1.161, (0.934, 1.388)	-	−0.142 (−0.370, 0.086)	-
50	0.94	-	1.052 (0.833, 1.271)	-	−0.036 (−0.234, 0.163)	-
60	0.88	-	0.847 (0.591, 1.104)	-	0.133 (−0.075, 0.341)	-
70	0.95	-	0.936 (0.768, 1.104)	-	0.055 (−0.063, 0.173)	-
80	0.93	-	0.884 (0.694, 1.074)	-	0.070 (−0.043, 0.183)	-
90	0.92	-	1.004 (0.766, 1.243)	-	0.003 (−0.109, 0.114)	-
100	0.85	-	1.004 (0.658, 1.351)	-	0.001 (−0.096, 0.097)	-
Full	0.99	-	1.017 (0.992, 1.042)	-	−0.003 (−0.021, 0.015)	-

*%*, percentage of one repetition maximum; CI, confidence intervals; MV, mean velocity; PV, peak velocity. If the 95% confidence interval for the intercept does not include 0, then fixed bias is present (*); if the 95% confidence interval for the slope does not include 1, then proportional bias is present (†).

**Table 4:** Least products regression for four devices in the power clean in comparison to 3D motion capture.

Load (%)	R^2^		Slope (95% CI)		Intercept (95% CI)	
	MV	PV	MV	PV	MV	PV
Gymaware						
40	0.93	0.91	1.165 (0.913, 1.417)	1.067 (0.787, 1.348)	−0.254 (−0.598, 0.090)	−0.172 (−0.841, 0.498)
50	0.95	0.93	0.944 (0.768, 1.120)	1.042 (0.806, 1.279)	0.042 (−0.192, 0.277)	−0.110 (−0.660, 0.441)
60	0.95	0.95	0.900 (0.731, 1.068)	0.974 (0.798, 1.150)	0.096 (−0.119, 0.312)	0.044 (−0.347, 0.435)
70	0.78	0.95	0.788 (0.443, 1.133)	1.061 (0.869, 1.253)	0.235 (−0.180, 0.650)	−0.133 (−0.545, 0.280)
80	0.86	0.91	0.831 (0.553, 1.110)	1.058 (0.801, 1.314)	0.167 (−0.148, 0.482)	−0.119 (−0.641, 0.403)
90	0.42	0.86	0.553 (0.023, 1.082)	0.949 (0.641, 1.257)	0.463 (−0.087, 1.014)	0.110 (−0.480, 0.700)
100	0.64	0.86	0.848 (0.334, 1.361)	0.879 (0.589, 1.170)	0.140 (−0.358, 0.368)	0.243 (−0.275, 0.762)
Full	0.94	0.96	0.956 (0.902, 1.009)	0.967 (0.918, 1.017)	0.032 (−0.030, 0.94)	0.069 (−0.036, 0.174)
PUSH Body						
40	0.38	0.27	0.713 (−0.021, 1.448)	0.655 (−0.229, 1.540)	0.431 (−0.498, 1.360)	1.213 (−0.351, 2.777)
50	0.50	0.43	0.485 (0.088, 0.881) †	0.663 (0.041, 1.285)	0.702 (0.214, 1.191) *	1.173 (0.102, 2.244) *
60	0.50	0.24	0.529 (0.103, 0.955) †	0.629 (−0.279, 1.537)	0.625 (0.123, 1.127) *	1.164 (−0.332, 2.661)
70	0.66	0.43	0.407 (0.167, 0.647) †	0.641 (0.043, 1.239)	0.703 (0.461, 0.999) *	1.149 (0.212, 2.086) *
80	0.54	0.27	0.274 (0.067, 0.481) †	0.426 (−0.140, 0.992) †	0.823 (0.607, 1.039) *	1.414 (0.589, 2.239) *
90	0.61	0.60	0.341 (0.118, 0.565) †	0.609 (0.201, 1.017)	0.719 (0.508, 0.930) *	1.126 (0.589, 1.663) *
100	0.34	0.66	0.327 (−0.048, 0.702) †	0.803 (0.329, 1.277)	0.677 (0.350, 1.005) *	0.833 (0.254, 1.412) *
Full	0.72	0.65	0.694 (0.591, 0.797) †	0.808 (0.664, 0.951) †	0.412 (0.298, 0.525) *	0.884 (0.663, 1.105) *
PUSH Bar						
40	0.62	0.59	0.923 (0.334, 1.512)	0.515 (0.166, 0.863) †	0.302 (−0.357, 0.961)	1.329 (0.617, 2.042)
50	0.54	0.11	0.502 (0.121, 0.882) †	0.256 (−0.331, 0.843) †	0.755 (0.342, 1.168)	1.807 (0.652, 2.961)
60	0.50	0.68	0.662 (0.126, 1.119)	1.070 (0.478, 1.662)	0.568 (0.017, 1.119)	0.216 (−0.883, 1.316)
70	0.24	0.08	0.227 (−0.102, 0.556) †	0.140 (−0.256, 0.535) †	0.951 (0.611, 1.291)	1.889 (1.148, 2.629)
80	0.35	0.06	0.242 (−0.026, 0.510) †	0.128 (−0.281, 0.537) †	0.872 (0.610, 1.134)	1.808 (1.089, 2.527)
90	0.41	0.26	0.395 (0.010, 0.780) †	0.391 (−0.144, 0.926) †	0.703 (0376, 1.031)	1.349 (0.562, 2.137)
100	0.23	0.60	0.286 (−0.138, 0.710) †	0.579 (0.197, 0.961) †	0.725 (0.372, 1.077)	0.966 (0.407, 1.525)
Full	0.62	0.48	0.765 (0.621, 0.910) †	0.536 (0.400, 0.672) †	0.414 (0.270, 0.558)	1.169 (0.925, 1.412)
Bar Sensei						
40	0.82	0.47	0.761 (0.478, 1.045)	0.591 (0.079, 1.103)	0.218 (−0.198, 0.634)	0.753 (−0.650, 2.156)
50	0.82	0.22	0.621 (0.386, 0.856) †	0.428 (−0.222, 1.077)	0.439 (0.113, 0.765) *	1.212 (−0.455, 2.879)
60	0.73	0.61	0.520 (0.262, 0.777) †	0.623 (0.218, 1.028)	0.545 (0.197, 0.894) *	0.706 (−0.269, 1.681)
70	0.04	0.50	0.081 (−0.243, 0.405) †	0.502 (0.095, 0.910) †	1.085 (0.693, 1.478) *	0.990 (0.047, 1.934) *
80	0.07	0.69	0.110 (−0.198, 0.417) †	0.582 (0.267, 0.897) †	0.985 (0.645, 1.326) *	0.781 (0.102, 1.460) *
90	0.18	0.84	0.213 (−0.151, 0.577) †	0.678 (0.442, 0.913) †	0.821 (0.449, 1.193) *	0.592 (0.128, 1.055) *
100	0.02	0.57	−0.071 (−0.460, 0.318) †	0.507 (0.145, 0.869) †	1.029 (0.657, 1.400) *	0.866 (0.190, 1.542) *
Full	0.73	0.74	0.569 (0.486, 0.652) †	0.608 (0.522, 0.693) †	0.479 (0.377, 0.580) *	0.726 (0.528, 0.924) *

*%* percentage of one repetition maximum; CI, confidence intervals; MV, mean velocity; PV, peak velocity. If the 95% confidence interval for the intercept does not include 0, then fixed bias is present (*); if the 95% confidence interval for the slope does not include 1, then proportional bias is present (†).
